# Antibacterial and Antifungal Activity of Propyl-Propane-Thiosulfinate and Propyl-Propane-Thiosulfonate, Two Organosulfur Compounds from *Allium cepa*: In Vitro Antimicrobial Effect via the Gas Phase

**DOI:** 10.3390/ph14010021

**Published:** 2020-12-29

**Authors:** Antonio Sorlozano-Puerto, Maria Albertuz-Crespo, Isaac Lopez-Machado, Lidia Gil-Martinez, Juan Jose Ariza-Romero, Alba Maroto-Tello, Alberto Baños-Arjona, Jose Gutierrez-Fernandez

**Affiliations:** 1 Department of Microbiology, School of Medicine and PhD Program in Clinical Medicine and Public Health, University of Granada-ibs, Avda. de la Investigación, 11, 18016 Granada, Spain; asp@ugr.es (A.S.-P.); albertuzmaria@correo.ugr.es (M.A.-C.); isloma@correo.ugr.es (I.L.-M.); 2 DMC Research Center, Camino de Jayena, 82, 18620 Alhendín, Spain; lidiagm@domca.com (L.G.-M.); jariza@dmcrc.com (J.J.A.-R.); albamaroto@dmcrc.com (A.M.-T.); abarjona@domca.com (A.B.-A.); 3 Laboratory of Microbiology, Virgen de las Nieves University Hospital-ibs, Avda. de las Fuerzas Armadas, 2, 18012 Granada, Spain

**Keywords:** propyl-propane-thiosulfinate, propyl-propane-thiosulfonate, antibacterial activity, antifungal activity, vapor

## Abstract

Propyl-propane thiosulfinate (PTS) and propyl-propane thiosulfonate (PTSO) are two volatile compounds derived from *Allium cepa* with a widely documented antimicrobial activity. The aim of this study was to evaluate their anti-candidiasis activity and the ability of its gaseous phase to inhibit bacterial and yeast growth in vitro. The minimum inhibitory concentration of various antifungal products (including PTS and PTSO) was determined versus 203 clinical isolates of *Candida* spp. through broth microdilution assay. Additionally, the antimicrobial activity through aerial diffusion of PTS and PTSO was evaluated over the growth of a collection of bacteria and yeasts cultivated in agar plates. All yeasts were susceptible to the antifungals tested, except *C. glabrata* and *C. krusei*, that showed azole resistance. PTSO (MIC_50_ and MIC_90_ ranged from 4 to 16 mg/L and 8 to 32 mg/L, respectively) was significantly more active against yeasts than PTS (MIC_50_ and MIC_90_ ranged from 16 to 64 mg/L and 32 to 64 mg/L). Values were higher than those obtained for antifungal drugs. Gaseous phases of PTS and PTSO generated growth inhibition zones whose diameters were directly related to the substances concentration and inversely related to the microbial inoculum. The quantification of PTS and PTSO levels reached in the growth media through aerial diffusion displayed a concentration gradient from the central zone to the periphery. Only *P. aeruginosa* ATCC 27853 showed resistance, while yeasts (*C. albicans* ATCC 200955 and *C. krusei* ATCC 6258) presented the higher susceptibility to both compounds. These results suggest that PTS and PTSO display antibacterial and anti-candidiasis activity in vitro through aerial diffusion, having potential use in human therapy.

## 1. Introduction

In recent years, the antioxidative, hypolipidemic, hypocholesterolemia, antihypertensive, heart-protective, antithrombotic, anticancer, anti-inflammatory, immunomodulatory, and antimicrobial activities of different organosulfur products, such as thiosulfinates and thiosulfonates obtained from garlic (*Allium sativum*) and onion (*Allium cepa*), especially allicin (diallyl thiosulfinate), have been thoroughly studied [1,2,3].

While the precise mechanism of action has not yet been discovered, the main antimicrobial effect of these *Allium*-derived compounds has been reported to be due to its chemical reaction with thiol groups present in the main enzymes of the microbial metabolism, such as succinate dehydrogenase, alcohol dehydrogenase, thioredoxin reductase and ureases among others, via thiol-disulfide exchange reaction [4,5,6]. Additionally, they can react with thiols, such as glutathione, increasing the oxidized glutathione rate into a range that induces oxidative stress and cellular apoptosis [7]; they can interact with enzymes taking part of the microbial system of acetyl-CoA blocking the incorporation of acetate into fatty acids and inhibiting the development of a phospholipid bilayer of the membranes [5]; and they can inhibit RNA polymerase and, therefore, block the total synthesis of microbial RNA [8].

In onion, the most common sulfur compounds are isoalliin (*S*-propenyl-l-cysteine sulfoxide), methiin (*S*-methyl-l-cysteine sulfoxide) and propiin (*S*-propyl-l-cysteine sulfoxide). Propiin changes into propyl-propane thiosulfinate (PTS) due to the action of aliinase [9]. Although it is more stable than allicin, PTS is also a labile compound that, through dismutation or disproportionation reactions, changes into dipropyl disulfide and propyl-propane thiosulfonate (PTSO) [10].

In a previous study we compared the in vitro antibacterial activity of PTS and PTSO with other antibiotics. Both molecules showed broad spectrum antibacterial activity against multiresistant bacteria isolated from human clinical samples. These results contribute to the development and potential use of these compounds against human infections, such as oral, gastrointestinal, or skin infections, as well as for the treatment of urinary tract infections [11].

Candiduria is a common finding in immunosuppressed and hospitalized patients, which determines the clinical relevance of its detection and treatment. In recent decades, a remarkable increase of opportunistic candidiasis infections has been described, especially those affecting urinary tract produced by *C. albicans*, the most common among them. Nevertheless, an increase in the incidence of other different species of *Candida* has been described, some of them characterized by a higher resistance to the most common antifungals used in humans [12,13]. Therefore, in the same way as with bacteria, it would be interesting to compare the in vitro anticandidal activity of PTS and PTSO with that of other existing antifungals for potential application in medical therapy.

Both substances are volatile. Therefore, the study of their antimicrobial capacity through their gaseous phase would have great interest for the valorization of their possible use in the treatment of susceptible respiratory pathogens if PTS and PTSO reach appropriate concentrations in pulmonary epithelium administered by inhalation [3,14].

For all these reasons, the aim of this study was to evaluate anti-candidiasis activity of PTS and PTSO in vitro and the capacity of its gaseous phase to inhibit the growth of different bacteria and yeasts.

## 2. Results

### 2.1. Antifungal Susceptibility

The identification of the 203 clinical isolates of *Candida* spp. was as follows: *C. albicans* (*n* = 83), *C. glabrata* (*n* = 73), *C. krusei* (*n* = 12) and *C. tropicalis* (*n* = 35). A summary of the antifungal susceptibility data is presented in Table 1. All the isolates of *C. glabrata* and *C. krusei* were resistant to fluconazole. *C. glabrata* was also resistant to voriconazole. In the other cases, the isolates were susceptible to the assayed antifungals. Amphotericin B significantly showed more activity against *C. albicans* and *C. tropicalis* than against the two other species (*p* < 0.001), while *C. glabrata* was the species with the lowest MIC values (*p* < 0.001) to echinocandins (anidulafungin, micafungin, and caspofungin).

The behavior of PTS and PTSO was quite homogeneous, regardless of the analyzed species (Table 1). MIC_50_ and MIC_90_ values of PTS ranged from 16 to 64 mg/L for the first and from 32 to 64 mg/L for the second. The MFC_50_ and MFC_90_ ranged from 32 to 128 mg/L and 128 mg/L, respectively. On the other hand, the values of MIC_50_ and MIC_90_ of PTSO ranged from 4 to 16 mg/L and 8 to 32 mg/L, while MFC_50_ y MFC_90_ ranged from 32 to 128 mg/L and from 64 to 128 mg/L, respectively. Considering all of the clinical isolates of *Candida* spp., the data indicated that PTSO was significantly more active than PTS (*p* < 0.001) and showed the fungicidal activity of these compounds since MIC and MFC values differed in only one dilution for both PTS (MIC_50_ = 32 mg/L and MFC_50_ = 64 mg/L; MIC_90_ = 64 mg/L and MFC_90_ = 128 mg/L) and PTSO (MIC_50_ = 16 mg/L and MFC_50_ = 32 mg/L; MIC_90_ = 32 mg/L and MFC_90_ = 64 mg/L).

### 2.2. Antimicrobial Activity of Vapor

**Figure 1:**
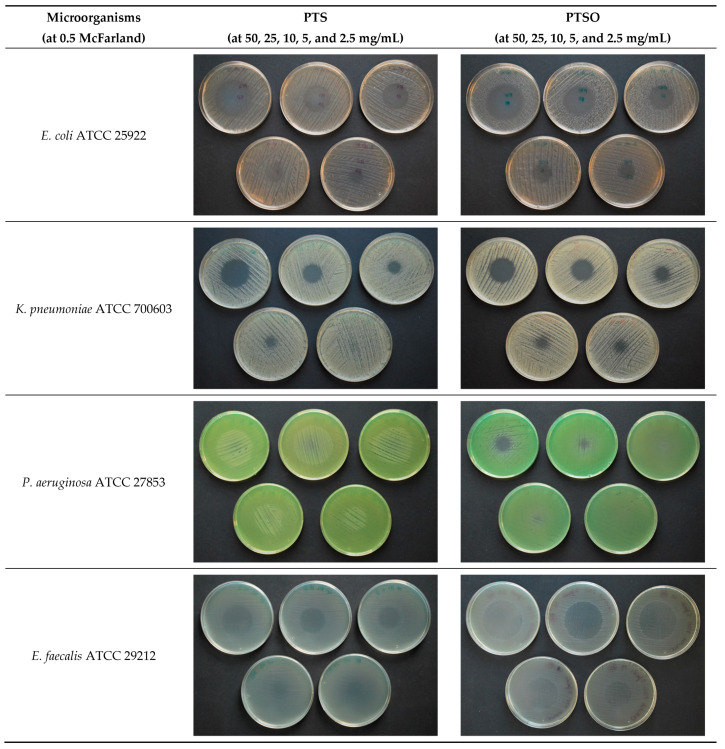

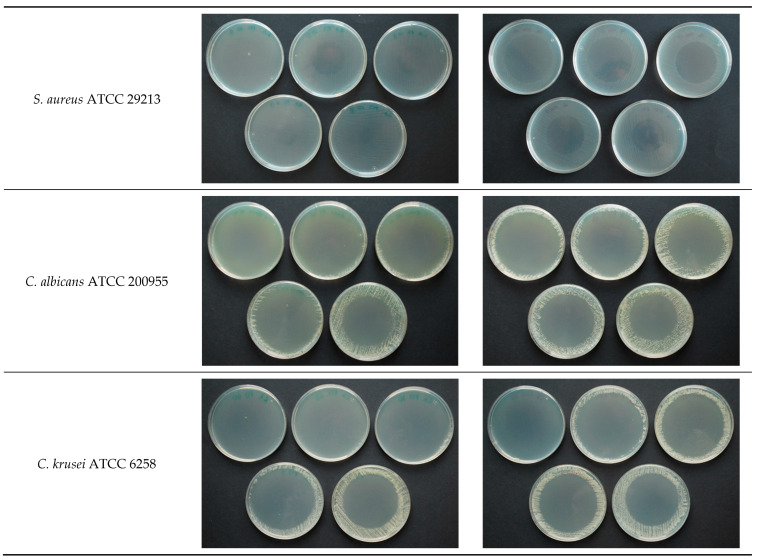
Antimicrobial activity of PTS and PTSO via the gas phase. Photograph showing halos of inhibition at doses of 50 mg/mL, 25 mg/mL, 10 mg/mL, 5 mg/mL, and 2.5 mg/mL (from left to right and from top to bottom). Although assays were carried out at 0.5, 1, and 2 McFarland turbidity, only the results at 0.5 McFarland are shown.

PTS and PTSO inhibited growth in six of the seven microorganisms tested in the present study through its gaseous phase without coming into contact with the medium and thus, with the microorganism, except for its aerial diffusion. The vapor produced by both substances reaches the agar medium, inhibiting microbial growth in a circular area above the drop placed in the lid of the Petri dish (Figure 1). The absence of any microbial growth in the inhibition area suggests a predominant biocidal effect of these substances. Only *P. aeruginosa* ATCC 27853 showed resistance to both compounds, showing absence of inhibition halo in the majority of the concentrations and bacterial inocula used. In the case of this bacterium, halos with a diameter below 10 mm were observed only when PTSO was used at a concentration of 50 mg/mL and 25 mg/mL against a bacterial inoculum at 0.5 of McFarland scale.

**Figure 2:**
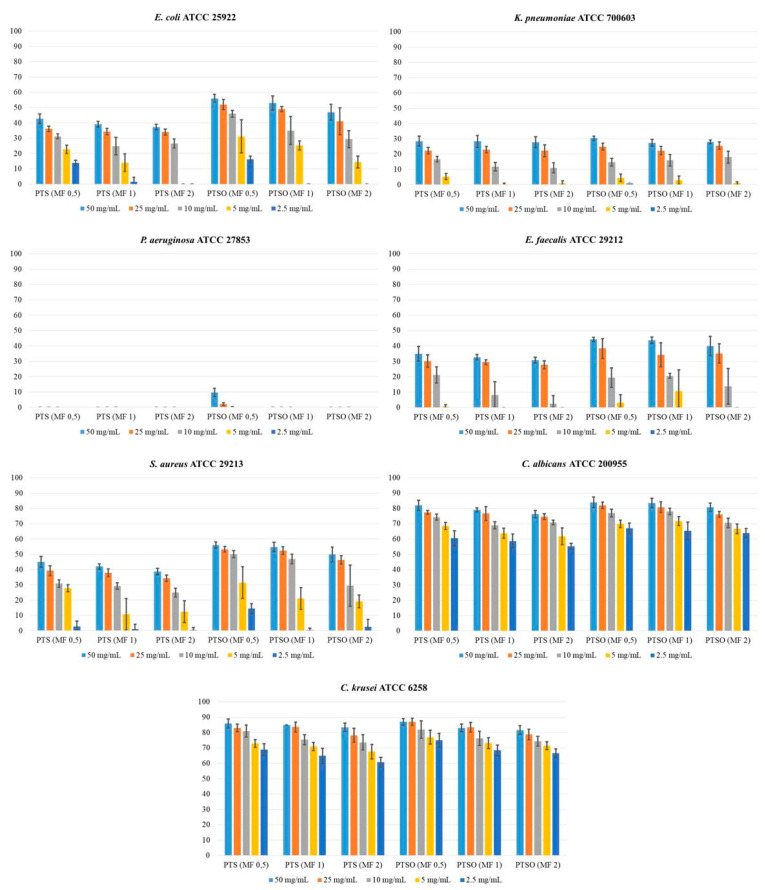
Average value ± standard deviation (in mm) of the growth inhibition halos for each concentration of PTS and PTSO, in the different inocula (0.5, 1, and 2 of McFarland) and for the different microorganisms. Diameters from the inhibition of growth zones were directly related to the concentration of PTS or PTSO used, and inversely related to the microbial inoculum used. Greater halos were observed in the case of yeasts. The diameters of the growth inhibition halos produced via the gas phase of PTSO were significantly larger than those obtained from PTS.

As it is shown in Figure 2 and Table 2, diameters from the inhibition of growth zones were directly related to the concentration of PTS or PTSO used, and inversely related to the microbial inoculum used: the higher the concentration of PTS or PTSO were and the lower the inoculum of microorganisms were, the larger the diameter of the halo was (*p* < 0.001). Greater halos and, consequently an increased susceptibility to these compounds were observed in the case of yeasts (*C. albicans* ATCC 200955 and *C. krusei* ATCC 6258).

Considering the set of microorganisms used in this study (with the exception of *P. aeruginosa* ATCC 27853 due to the demonstrated resistance to both compounds), the antimicrobial activity of PTSO was significantly higher than that of PTS, since, for the same microbial inoculum and substance concentration, the diameters of the growth inhibition halos produced by the gas phase of PTSO were significantly larger than those obtained from PTS (*p* < 0.01, in all cases).

Table 3 shows the concentration of PTS and PTSO detected in the Mueller–Hinton medium by HPLC-UV. The gas phase of both compounds, deposited on the lid of the Petri dish, reached the culture medium being able to be detected and quantified. The highest concentration in all cases was reached in sample 1, which coincided with the center of the plate, located in the most vertical position just above the drop deposited on the lid. As the sample moved away from the center (samples from 2 to 6), the concentration decreased, creating a gradient from the central area of the plate (highest concentration) towards its periphery (lowest concentration). In some cases, either because the concentration of the drop was small (2.5 mg/mL) and/or because the distance to the center was higher (sample 6), the concentration reached in the medium could not be quantified because it was below the limit of detection of the HPLC-UV technique.

**Figure 3:**
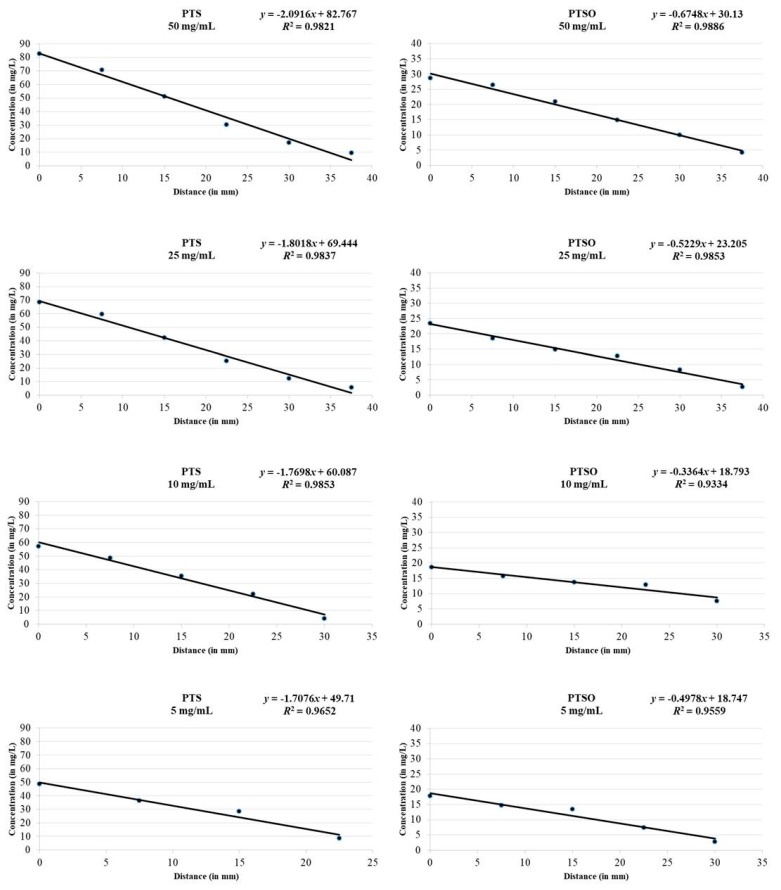
Relationship between the distance from the center of the Petri dish and the concentration reached via the gas phase at a defined point of the Mueller–Hinton medium for each concentration of PTS and PTSO deposited on the lid of the Petri dish. There is an inverse linear relationship between the distance to the center of the culture medium and the concentration reached via the gas phase at a given point.

From the results shown in Table 3, the linear regression lines were drawn (Figure 3), relating the distance to the center of the plate (measured in mm) to the concentration that PTS or PTSO reached at a given point (in mg/L) according to each of the concentrations of the drop deposited on the lid. As it can be seen, there was a high correlation in all cases, which determined the quality of the fit. 

Theoretical concentration of PTS and PTSO reached at the limit of the microbial growth inhibition zone for each microorganism and inoculum concentration is shown in Table 4. This concentration was determined by measuring the radius (in mm) of the inhibition areas obtained for each microorganism and inoculum concentration and extrapolating the results in the linear regression line for the corresponding concentrations of PTS and PTSO showed in Figure 3. The concentrations of PTSO at which microbial growth was inhibited were lower than those of PTS (*p* < 0.01), showing a greater antimicrobial activity. Furthermore, the inhibition of yeast growth compared to that of the bacteria occurred with lower concentrations, with either of the two compounds. 

## 3. Discussion

### 3.1. Antifungal Susceptibility

Invasive fungal diseases of nosocomial origin or associated to health care, especially those caused by *Candida* spp., have become a major health problem as they are associated with high rates of morbidity and mortality. Although candida urinary tract infections and vulvovaginal infections are, a priori, milder processes, they have a higher incidence among the population and can be the origin of more serious and disseminated infections in patients with underlying diseases [15]. Similarly, yeast colonization of the skin and mucosal surfaces is also a risk factor for the development of invasive candidiasis in patients, especially those admitted in the intensive care unit as a consequence of risk factors such as handling by colonized healthcare personnel, central venous and urinary catheters, use of broad-spectrum antibiotics, prolonged lengths of stay, mechanical ventilation, parenteral feeding, etc. [16].

On the other hand, alongside the increase of the prevalence of yeast infections by genus *Candida*, a significant increase in the rates of resistance to antifungals commonly used in human clinics (mainly polyenes, azoles, and echinocandins) is currently described both for *C. albicans* as in other non-*albicans* species. Therefore, it is difficult to establish a preventive and therapeutic approach to these infections, which makes advisable to research new alternative therapies to conventional treatments with different and/or synergistic mechanisms of fungicide action and fewer side effects [17,18,19,20].

In this context, the thiosulfinates derived from *Allium* spp., such as allicin, have demonstrated broad antifungal activity in numerous in vitro studies against yeasts of the genus *Candida* [21,22,23]. In the present work, the MICs obtained for antifungals were similar to those obtained in previous research, both in our geographical area [24,25] and in more remote areas [19,26,27,28,29]. The lowest MICs were obtained for *C. albicans* and *C. tropicalis*; the highest for *C. glabrata* and *C. krusei*, especially in the case of azoles, which indicates that *Candida* species remain susceptible to commonly used antifungals and do not represent a problem for the therapeutic approach. The usual resistance of *C. glabrata* and *C. krusei* to azoles would be an exception.

Similarly, our results demonstrate that PTS and PTSO have a significant antifungal activity against different isolates of *Candida* spp. from human clinical samples, although their activity is not as strong as that of some antifungals, such as amphotericin B, echinocandins or azoles, especially if we consider the most susceptible species to them. In addition, our findings related to the antifungal effects of other organosulfur compounds against yeast isolates are in compliance with those already described in the literature [21,30,31].

In order to explain the antifungal effect of these molecules, different mechanisms of action have been proposed. The most directly associated to cell damage and decreased growth capacity of the fungus are the ability to modify essential enzymes in fungal metabolism [32], induce oxidative stress [7], alter lipids by damaging the integrity of cell membranes (including cytoplasmatic organelles, such as mitochondria or vacuoles) [33] and modify the expression of some genes [34].

Even if our preliminary results provide useful information about the potential use of PTS and PTSO for the prevention or treatment of candidiasis infections caused by multidrug resistant yeasts, further research is needed to demonstrate the effectiveness of these compounds with a wider group of fungi and in vivo models [35]. Even though these results may support their therapeutic use, the absence of cut-off points defined by international committees for this kind of substances does not allow relevant conclusions to be drawn. Further investigation regarding their pharmacokinetic and toxicological characteristics is required before considering safe clinical use.

### 3.2. Antimicrobial Activity of Vapor

One of the main characteristics of the organosulfur compounds obtained from plants of the genus *Allium* is their volatility, which is the main reason of the characteristic aroma that these plants exude, especially when they are mashed or crushed. Given the scarcity of volatile antimicrobials available for clinical use in humans, these molecules could be considered an alternative (alone or in combination with other antimicrobials) for the treatment of lung infections via inhalation, instead of oral or parenteral administration [3]. However, there are still few studies evaluating their antimicrobial capacity through their gas phase and their potential applicability for the treatment of infectious diseases [3,14,36].

In the present work, PTS and PTSO showed high bactericidal and fungicidal activity through their gas phase, inhibiting the growth of six of the seven microorganisms assayed (*E. coli* ATCC 25922, *K. pneumoniae* ATCC 700603, *E. faecalis* ATCC 29212, *S. aureus* ATCC 29213, *C. albicans* ATCC 200955 and *C. krusei* ATCC 6258, but not *P. aeruginosa* ATCC 27853), which might suggest that these substances are likely to be less active against *P. aeruginosa* than against other pathogens. Various studies have demonstrated that, against these bacteria and other related ones, very high concentrations of these compounds should be used to inhibit bacterial growth, which may not be feasible from a therapeutic point of view [11,37,38].

However, the microbicidal activity of these compounds was not the same against both types of microorganisms. The antifungal effect was higher than the antibacterial effect (the growth inhibition halos were significantly higher in *C. albicans* ATCC 200955 and *C. krusei* ATCC 6258 in comparison to those obtained against bacteria). The higher activity of these compounds on yeasts is also observed if we compare the results obtained in the present work on antifungal susceptibility, in terms of MIC, with a previous work of our group with clinical bacterial isolates from human origin: MIC_50_ and MIC_90_ values of PTS and PTSO are lower against yeasts than against bacteria [11]. According to some studies, it is possible that the presence of a fungal cell wall, more permeable to these compounds than the peptidoglycan wall of the bacteria, allows this cytotoxic effect at lower doses [39]. On the other hand, the gas phase of PTSO was more active than that of PTS against all the microorganisms evaluated (with the exception of *P. aeruginosa*), since the growth inhibition halos were significantly higher for PTSO than for PTS in all cases. In fact, the higher antimicrobial activity of PTSO in comparison to PTS has already been described previously by our group [11].

As shown by Leontiev et al. [14], despite of the lack of direct contact of the organosulfur compounds with the agar and with the microorganism itself, when the antimicrobial effect via the gas phase is studied, it is worth noting how clear are the microbial growth inhibition halos and how well defined are the edges. His interpretation, as well as ours, is that this could be due to the existence of a concentration gradient from the closest area of the agar to the drop, precisely at its zenith, to the periphery. In our study, the quantification of the concentration of PTS and PTSO in the agar allowed to confirm this suspicion, showing that there is an inverse linear relationship between the distance to the center of the culture medium and the concentration reached via the gas phase at a given point.

From the results obtained in the present study, as well as from previous works [3,14,40] it can be deduced that organosulfur compounds derived from *Allium* spp., such as PTS and especially PTSO could be used for the treatment of lung infections, due to its high volatility by inhalation, producing an effect directly on the lung. An advantage that would facilitate their clinical use is that these substances are perceived as innocuous as they are present naturally in food such as onion, widely consumed and included in the diet in most cultures. Among the disadvantages, we could highlight the need to improve the extraction procedures in order to preserve the biological properties of these substances avoiding their loss as a consequence of events such as heating, long-term preservation, etc. In addition, there is a need to diminish the strong impact of their smell and aroma.

However, since the microbicidal effect is directly proportional to the concentration of PTS and PTSO used, a definitive conclusion on the efficacy of this treatment cannot be drawn without evaluating the potential toxicity on human lung cells. It may be possible that the dose required to exert the expected effect in vivo is so high that it produces undesirable toxic consequences. As indicated by Reiter et al. [3], the use of lower and less toxic concentrations concomitantly with other antibiotics or antifungals could be considered if a synergistic effect is demonstrated. In this sense, it has been proven that thiosulfinates have a synergistic effect with antibiotics, such as ampicillin, piperacillin-tazobactam, levofloxacin, gentamicin, amikacin, azithromycin, vancomycin, doxycycline, or polymyxin B [41,42,43,44,45] and with antifungals such as azoles [22,46] or amphotericin B [21]. The potential clinical usefulness of these substances at low concentrations, via inhalation and in combination with other antimicrobials, requires further studies in vitro and with animal and/or human models.

Among the main limitations of our study, was that the methodologies used, both for the study of the antifungal effect of PTS and PTSO, as well as for the antimicrobial effect that their gas phase could exert, are not standardized. Therefore, it is not possible to establish a direct correlation between the results obtained and the potential human therapeutic use. Although their antimicrobial activity seems obvious as numerous studies have shown, international committees for the study of antimicrobial susceptibility have not yet defined cut-off points for these compounds. Thus, no final conclusion can be drawn. Furthermore, the present work has not evaluated the volatility of the organosulfur derivatives and, therefore, the possible loss of activity of PTS and PTSO over time or with increasing temperature, which other authors have described previously [36]. These factors could reduce their clinical usefulness. Finally, further studies should be focused on standardizing the methods to evaluate the antimicrobial activity, understanding how these substances are distributed or removed from the organism, determining the administration routes, and evaluating their effect in different dosages, organs and systems and evaluating the safety of their administration in humans, before safe clinical use is considered.

## 4. Materials and Methods

### 4.1. Antifungal Susceptibility Testing

#### 4.1.1. Antifungals, PTS, and PTSO

Amphotericin B, anidulafungin, micafungin, caspofungin, fluconazole and voriconazole were purchased from Sigma-Aldrich (Madrid, Spain). PTS and PTSO are organosulfur compounds present in onion extracts (AlioCare^TM^) which were supplied with high purity (97%) by Enzim-Orbita Agroalimentares LDA (Tavira, Portugal) and dissolved in polysorbate-80 to a final concentration of 500,000 mg/L.

#### 4.1.2. Candida Isolates and Identification

Two hundred and three clinical isolates of *Candida* spp. obtained from urine samples processed in the Laboratory of Microbiology of the Virgen de las Nieves University Hospital (Granada, Spain) were selected. CHROMagar Orientation medium (Becton Dickinson, Franklin Lakes, NJ, USA) was used for the growth of isolates. All colonies with yeast compatible morphology were subcultured using a CHROMagar Candida medium (Becton Dickinson). Species were identified using filamentation test and ASM Vitek system (bioMérieux, Madrid, Spain) or MALDI Biotyper system (Bruker Daltonics, Billerica, MA, USA). All isolates were stored at −40° C until the susceptibility study.

#### 4.1.3. In Vitro Antifungal Assay

Standard broth microdilution method was carried out according to the guidelines of the Clinical and Laboratory Standards Institute (CLSI) [47]. A series of two-fold final dilutions of each drug, PTS, and PTSO were prepared in RPMI-1640 medium with L-glutamine but without bicarbonate. Glucose was added to a final concentration of 0.2% and pH was adjusted to 7.0 with acid morpholine propane sulfonic (0.165 M) buffer.

First, all isolates were subcultured onto Sabouraud dextrose agar. Twenty-four hours after the incubation, standard 0.5 McFarland fungal suspensions were prepared with saline solution. Microdilution testing was carried out in 96-well, flat-bottom microtiter plates with a final concentration of the yeast cell suspension equal to 1–5 × 10^3^ cells/mL in each well. Each plate contained 10 serial two-fold dilutions of each antifungal compound, PTS or PTSO. The range of concentrations (in mg/L) assayed for each compound were as follows: amphotericin B (0.03–16), anidulafungin (0.008–4), micafungin (0.008–4), caspofungin (0.008–4), fluconazole (0.125–64) and voriconazole (0.008–4). The concentration ranges of both PTS and PTSO were 0.25–128 mg/L. The positive controls (yeast suspension without antifungal) and negative control (RPMI medium) were added in the last two wells of the plate.

Microtiter plates were incubated at 35 °C and the minimum inhibitory concentration (MIC) values were assessed visually after 24 h (48 h in case of azoles). For amphotericin B, the MIC was determined as the lowest concentration of drug which produced a total inhibition of visual growth. For azoles and echinocandins, the MICs were defined as the lowest concentration of drug that produced ≥50% reduction of visual growth in comparison with the growth of control wells.

The clinical isolates were considered to be susceptible (S), intermediate (I) or susceptible dose-dependent (SDD, only for fluconazole), or resistant (R) to anidulafungin, micafungin, caspofungin, fluconazole and voriconazole according to the recommendations of the CLSI [48]. For amphotericin B, European Committee on Antimicrobial Susceptibility Testing (EUCAST) guidelines were followed [49]. CLSI clinical breakpoints for the susceptibility patterns of *C. albicans*, *C. tropicalis*, and *C. krusei* to anidulafungin, micafungin, and caspofungin were S ≤ 0.25 mg/L, I = 0.5 mg/L, and R ≥ 1 mg/L. Breakpoints for the susceptibility patterns of *C. glabrata* to anidulafungin and caspofungin were S ≤ 0.12 mg/L, I = 0.25 mg/L, and R ≥ 0.5 mg/L; and to micafungin were S ≤ 0.06 mg/L, I = 0.12 mg/L, and R ≥ 0.25 mg/L. CLSI clinical breakpoints for the susceptibility patterns of *C. albicans* and *C. tropicalis* to fluconazole were S ≤ 2 mg/L, SDD = 4 mg/L, and R ≥ 8 mg/L; and for *C. glabrata* SDD ≤ 32 mg/L, and R ≥ 64 mg/L. For voriconazole, S, I, and R breakpoints for *C. albicans* and *C. tropicalis* were ≤ 0.12 mg/L, 0.25–0.5 mg/L, and ≥ 1 mg/L, respectively, and for *C. krusei* ≤ 0.5 mg/L, 1 mg/L, and ≥ 2 mg/L, respectively. With regard to amphotericin B, the following cutoff values were used for all yeasts: S < 1 mg/L and R ≥ 1 mg/L. There are no cut-off points defined in CLSI or EUCAST for *C. krusei* to fluconazole neither *C. glabrata* to voriconazole. All isolates of *C. krusei* and *C. glabrata* were then considered resistant to fluconazole or voriconazole, respectively, regardless of the MICs. The values of MIC_50_ and MIC_90_ were determined as the lowest concentration of the antifungal at which 50% and 90% of the isolates were inhibited, respectively.

To determine the minimal fungicidal concentration (MFC), after mixing the contents of each well, 100 μL were inoculated onto a plate with Sabouraud dextrose agar and incubated at 35 °C for 48 h. The lowest concentrations that showed no growth after incubation gave the MFC value. MFC_50_ and MFC_90_ values were defined as the concentration of antifungal that kills 50% and 90% of the isolates, respectively.

Following the CLSI and EUCAST guidelines, *C. krusei* ATCC 6258 was used as quality control in the procedures.

### 4.2. Antimicrobial Activity of Gaseous PTS and PTSO

Seven microorganisms from the ATCC collection (American Type Culture Collection, Manassas, VA, USA) were used. *Escherichia coli* ATCC 25922, *Klebsiella pneumoniae* ATCC 700603, and *Pseudomonas aeruginosa* ATCC 27853 were used as representative Gram-negative bacteria. *Enterococcus faecalis* ATCC 29212 and *Staphylococcus aureus* ATCC 29213 were used as representative Gram-positive bacteria. Finally, *Candida albicans* ATCC 200955 and *Candida krusei* ATCC 6258 were used as representative yeast.

Bacteria and yeasts were grown over night at 36 ± 1 °C on sheep blood agar and Sabouraud dextrose agar plates, respectively. Colonies were suspended in 5 mL saline solution at 0.5, 1, and 2 McFarland turbidity. Bacteria were spread on Mueller-Hinton agar and yeast on Mueller–Hinton agar supplemented with 2% glucose. Drops of 20 μL of different concentrated PTS and PTSO solutions (50 mg/mL, 25 mg/mL, 10 mg/mL, 5 mg/mL, and 2.5 mg/mL) were placed in the center of a 9-cm diameter Petri dish lid and the solidified agar plates with bacteria or yeasts were placed inverted over the lid according to a previously described procedure [3,14]. Thus, the test solution and the agar itself did not come into contact except by diffusion through the air. After incubation for 24 h at 36 ± 1 °C the diameter of the inhibition zone was measured. Each trial was repeated 10 times.

**Figure 4:**
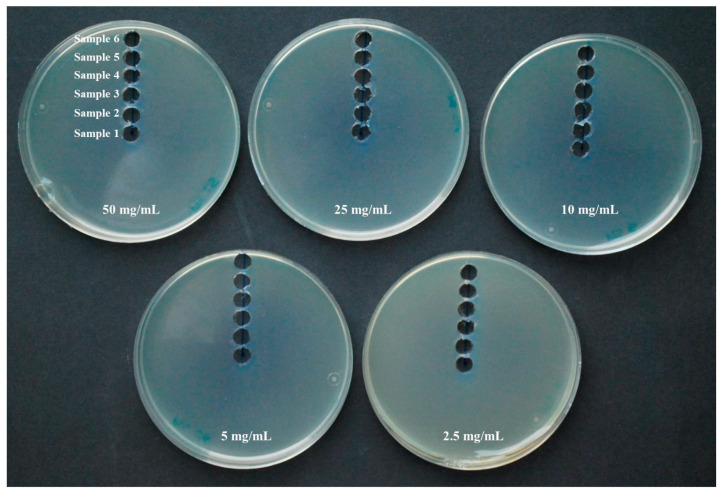
Procedure for obtaining samples from the Mueller–Hinton agar growth medium to establish the concentration of PTS and PTSO reached on it via the gas phase of both compounds by HPLC-UV. For each organosulfur compound and concentration, after incubation for 24 h at 36 ± 1 °C, samples with a diameter of 7.5 mm were extracted from the center of the plate to the periphery in order to determine the concentration of PTS and PTSO reached on the agar as a consequence of its evaporation.

In the same way as in the previous procedure but without the use of any microorganism, drops of 20 μL of different concentrations of PTS and PTSO solutions (50 mg/mL, 25 mg/mL, 10 mg/mL, 5 mg/mL and 2.5 mg/mL) were placed in the center of the Petri dishes lid and the solidified Mueller–Hinton agar plates were placed inverted over the lid. After incubation for 24 h at 36 ± 1 °C samples of the growth media with a diameter of 7.5 mm were extracted from the center of the plate to the periphery in order to determine the concentration of PTS and PTSO reached on the agar as a consequence of its evaporation (Figure 4). Each trial was repeated three times. The PTS and PTSO concentration achieved in each of the Mueller–Hinton agar samples was determined by high-performance liquid chromatography using a UV detector (HPLC-UV).

### 4.3. HPLC-UV Analysis

For the analysis of PTS and PTSO in agar samples, an Agilent 1260 Infinity HPLC (Agilent Technologies Inc., Waldbron, Germany) system was used. The system is equipped with an online degasser, an autosampler, a column thermostat, a diode array detector, and a quaternary pump. The technology used to determine PTS and PTSO was previously described by our group [50,51]. The analysis was carried out in a C18 column (Zorbax Eclipse Plus 50 mm × 4.6 mm, 1.8 μm). Solvents used were 30 mM perchloric acid and MeCN (solvent A and B, respectively) dissolved in water at a flow rate of 0.85 mL min^−1^. The injection volume was 10 μL and the gradient elution program was: 0 min, 50% B; 2.2 min, 50% B; 4.5 min, 100% B; 6.8 min, 100% B; 8 min, 50% B; 10.5 min, 50% B. The wavelength of detection was set at 200 nm. Agar samples were individually weighed and extracted in 500 μL of methanol through 5 min in vortex. The extract was filtered and directly injected into the HPLC-UV.

### 4.4. Statistical Analysis

In order to compare the distribution of MIC values of antifungals, PTS and PTSO in the different groups of yeasts studied, the Wilcoxon rank-sum test was used. The differences in the diameters of the growth inhibition halos and in the concentrations reached in the limits of the zone of microbial growth inhibition of the microorganisms after exposure to the gaseous phase of PTS and PTSO were compared using the Wilcoxon signed-rank test. *p*-values < 0.05 were considered statistically significant. Data analysis was performed using the software IBM SPSS Statistics, version 25.0. (IBM Corporation, Armonk, NY, USA).

## 5. Conclusions

PTS, and especially PTSO, showed antibacterial and anti-candida activity, even during their gaseous phase, which makes them potentially useful molecules for human therapy. However, it would be necessary to establish its efficacy in human trials, and to know the concentrations that they achieve in the lung tissue when they are administered by inhalation.

## Figures and Tables

**Table 1:** In vitro activity of different antifungal drugs, PTS, and PTSO against *Candida* spp.

Species of Yeasts (Number of Isolates)	Range of MIC (in mg/L)	MIC_50_ (in mg/L)	MIC_90_ (in mg/L)	Range of MFC (in mg/L)	MFC_50_ (in mg/L)	MFC_90_ (in mg/L)	Number of Resistant Isolates
***Candida albicans* (*n* = 83)**							
Amphotericin B	0.125–0.25	0.25	0.25	1–16	4	8	0
Anidulafungin	≤0.008–0.25	0.015	0.06	0.015–>4	0.25	1	0
Micafungin	≤0.008–0.25	0.03	0.125	0.03–4	0.5	2	0
Caspofungin	≤0.008–0.25	0.06	0.06	0.06–4	0.5	2	0
Fluconazole	≤0.125–2	0.25	2	8–>64	>64	>64	0
Voriconazole	≤0.008–0.06	≤0.008	≤0.008	0.03–0.5	0.5	0.5	0
PTS	16–64	32	32	16–>128	32	128	-
PTSO	8–64	16	32	8–128	32	64	-
***Candida glabrata* (*n* = 73)**							
Amphotericin B	0.06–1	0.25	0.5	1–>16	4	8	0
Anidulafungin	≤0.008–0.125	0.015	0.03	0.06–>4	0.25	0.5	0
Micafungin	≤0.008–0.06	≤0.008	0.03	0.008–4	0.06	0.25	0
Caspofungin	≤0.008–0.125	0.03	0.03	0.06–4	1	2	0
Fluconazole	0.25–≥64	4	≥64	16–>64	>64	>64	73
Voriconazole	0.03–≥4	0.5	≥4	2–>4	>4	>4	73
PTS	8–64	32	32	16–>128	64	128	-
PTSO	16–32	16	32	8–>128	32	64	-
***Candida krusei* (*n* = 12)**							
Amphotericin B	0.25–1	0.5	1	4–16	8	16	0
Anidulafungin	≤0.008–0.06	0.015	0.03	0.125–0.5	0.25	0.5	0
Micafungin	0.06–0.25	0.125	0.125	0.5–4	0.5	1	0
Caspofungin	0.06–0.25	0.125	0.25	1–4	1	2	0
Fluconazole	8–≥64	16	≥64	>64	>64	>64	12
Voriconazole	0.03–0.5	0.125	0.5	2–>4	>4	>4	0
PTS	4–32	16	32	32–>128	32	128	-
PTSO	4–16	4	8	64–>128	128	128	-
***Candida tropicalis* (*n* = 35)**							
Amphotericin B	≤0.03–1	0.125	0.25	2–16	2	8	0
Anidulafungin	≤0.008–0.25	0.06	0.25	0.015–2	1	2	0
Micafungin	≤0.008–0.25	0.06	0.25	0.008–2	0.5	2	0
Caspofungin	≤0.008–0.25	0.06	0.25	0.06–4	1	4	0
Fluconazole	≤0.125–2	≤0.125	1	8–>64	>64	>64	0
Voriconazole	≤0.008–0.06	≤0.008	0.015	0.03–0.5	0.5	0.5	0
PTS	32–64	64	64	64–>128	128	128	-
PTSO	4–32	16	32	4–128	32	64	-

MIC: minimum inhibitory concentration. MFC: minimum fungicidal concentration.

**Table 2:** Average diameter ± standard deviation (in mm) of the growth inhibition halos for the different PTS and PTSO concentrations, in each inoculum (0.5, 1, and 2 from McFarland) and for the different microorganisms.

Microorganisms	McFarland	PTS Concentration (Growth Inhibition in mm)					PTSO Concentration (Growth Inhibition in mm)				
		50 mg/mL	25 mg/mL	10 mg/m L	5 mg/mL	2.5 mg/mL	50 mg/mL	25 mg/mL	10 mg/mL	5 mg/mL	2.5 mg/mL
*E. coli* ATCC 25922	0.5	43 ± 3.2	36 ± 1.9	31 ± 1.6	23 ± 2.7	14 ± 1.6	56 ± 2.7	52 ± 3.3	46 ± 2.2	31 ± 10.8	16 ± 2.1
	1	39 ± 2.0	34 ± 2.1	25 ± 5.6	14 ± 5.7	1 ± 3.0	53 ± 4.5	49 ± 1.8	35 ± 9.2	25 ± 3.0	0
	2	37 ± 1.8	34 ± 2.0	27 ± 3.0	0	0	47 ± 5.1	42 ± 8.8	29 ± 5.5	14 ± 3.9	0
*K. pneumoniae* ATCC 700603	0.5	28 ± 3.3	22 ± 2.2	17 ± 1.8	5 ± 2.0	0	30 ± 1.3	25 ± 2.2	15 ± 2.5	5 ± 2.5	1 ± 0.7
	1	28 ± 3.9	23 ± 2.2	12 ± 2.8	0	0	27 ± 2.3	22 ± 2.7	16 ± 3.8	3 ± 2.7	0
	2	28 ± 3.6	22 ± 3.8	11 ± 3.3	1 ± 1.8	0	28 ± 1.2	26 ± 2.4	18 ± 3.9	1 ± 0.9	0
*P. aeruginosa* ATCC 27853	0.5	0	0	0	0	0	10 ± 2.7	2 ± 0.9	0	0	0
	1	0	0	0	0	0	0	0	0	0	0
	2	0	0	0	0	0	0	0	0	0	0
*E. faecalis* ATCC 29212	0.5	35 ± 4.6	30 ± 4.0	21 ± 5.2	1 ± 1.2	0	44 ± 1.3	38 ± 6.5	20 ± 6.3	3 ± 5.3	0
	1	33 ± 1.8	30 ± 1.6	8 ± 8.7	0	0	44 ± 2.1	34 ± 7.8	21 ± 1.6	11 ± 14.0	0
	2	31 ± 1.9	28 ± 2.7	3 ± 5.3	0	0	40 ± 6.3	35 ± 6.2	14 ± 11.6	0	0
*S. aureus* ATCC 29213	0.5	45 ± 3.6	39 ± 3.3	31 ± 2.4	28 ± 2.4	3 ± 3.5	56 ± 2.0	53 ± 1.8	50 ± 2.3	31 ± 10.4	14 ± 3.3
	1	42 ± 1.8	38 ± 2.5	29 ± 2.0	11 ± 10.3	1 ± 3.2	55 ± 3.1	52 ± 2.5	47 ± 3.2	21 ± 7.1	0
	2	39 ± 2.1	34 ± 2.1	25 ± 2.8	12 ± 7.1	1 ± 1.6	50 ± 4.9	46 ± 2.7	29 ± 13.5	19 ± 4.3	3 ± 4.8
*C. albicans* ATCC 200955	0.5	82 ± 3.4	77 ± 1.2	74 ± 1.9	69 ± 2.3	61 ± 4.9	84 ± 3.4	82 ± 2.2	77 ± 2.5	70 ± 2.4	67 ± 3.5
	1	79 ± 1.3	77 ± 4.5	69 ± 2.3	64 ± 3.3	59 ± 4.5	84 ± 3.2	81 ± 3.4	78 ± 2.1	72 ± 3.0	65 ± 5.8
	2	76 ± 2.5	75 ± 1.9	71 ± 1.5	62 ± 5.5	55 ± 1.9	81 ± 2.8	76 ± 1.9	71 ± 3.0	67 ± 3.2	64 ± 2.9
*C. krusei* ATCC 6258	0.5	86 ± 3.0	83 ± 2.5	81 ± 3.9	73 ± 2.5	69 ± 3.9	87 ± 2.1	87 ± 2.3	82 ± 5.6	77 ± 4.5	75 ± 4.5
	1	85 ± 0.0	84 ± 3.3	75 ± 3.2	71 ± 2.6	65 ± 5.0	83 ± 2.5	84 ± 3.2	76 ± 4.7	73 ± 3.6	69 ± 3.4
	2	84 ± 2.7	78 ± 4.5	74 ± 4.9	68 ± 4.7	61 ± 3.2	82 ± 2.8	79 ± 3.5	75 ± 3.2	72 ± 2.5	67 ± 2.8

**Table 3:** Concentration ± standard deviation (in mg/L) of PTS and PTSO reached in Mueller–Hinton agar for each of the samples, in relation to the initial concentration of each substance in the drop deposited on the lid of the Petri dish.

Organosulfur Compound	Initial Concentration	Sample 1	Sample 2	Sample 3	Sample 4	Sample 5	Sample 6
PTS	50 mg/mL	82.7 ± 0.1	70.8 ± 0.3	51.2 ± 0.1	30.2 ± 0.2	17.0 ± 0.3	9.4 ± 0.1
	25 mg/mL	68.6 ± 0.2	59.6 ± 0.1	42.4 ± 0.3	25.1 ± 0.2	12.4 ± 0.1	5.8 ± 0.2
	10 mg/mL	57.3 ± 0.2	48.6 ± 0.1	35.5 ± 0.1	22.1 ± 0.2	4.2 ± 0.1	<LD
	5 mg/mL	48.5 ± 0.2	36.4 ± 0.2	28.6 ± 0.3	8.5 ± 0.2	<LD	<LD
	2.5 mg/mL	<LD	<LD	<LD	<LD	<LD	<LD
PTSO	50 mg/mL	28.6 ± 0.2	26.3 ± 0.2	20.9 ± 0.2	14.8 ± 0.1	9.9 ± 0.4	4.2 ± 0.2
	25 mg/mL	23.4 ± 0.2	18.6 ± 0.2	14.8 ± 0.2	12.7 ± 0.2	8.2 ± 0.2	2.6 ± 0.1
	10 mg/mL	18.7 ± 0.1	15.8 ± 0.1	13.8 ± 0.1	12.9 ± 0.3	7.5 ± 0.1	<LD
	5 mg/mL	17.9 ± 0.1	14.8 ± 0.3	13.4 ± 0.2	7.5 ± 0.1	2.8 ± 0.3	<LD
	2.5 mg/mL	<LD	<LD	<LD	<LD	<LD	<LD

LD: Limit of detection of the HPLC-UV technique.

**Table 4:** Average value ± standard deviation (in mg/L) of the concentration of PTS and PTSO reached at the limit of the microbial growth inhibition zone.

Microorganism	McFarland	Limit of the Microbial Growth	PTS Concentration				PTSO Concentration			
			50 mg/mL	25 mg/mL	10 mg/mL	5 mg/mL	50 mg/mL	25 mg/mL	10 mg/mL	5 mg/mL
*E. coli* ATCC 25922	0.5	Radius	21.5	18	15.5	11.5	28	26	23	15.5
		Concentration	38.1 ± 3.3	36.9 ± 1.7	32.5 ± 1.4	30.3 ± 2.3	11.3 ± 0.9	9.5 ± 0.9	11.1 ± 0.4	11.0 ± 2.7
	1	Radius	19.5	17	12.5	7	26.5	24.5	17.5	12.5
		Concentration	41.9 ± 2.1	38.5 ± 1.9	38.1 ± 5.0	37.8 ± 4.9	12.2 ± 1.5	10.4 ± 0.5	12.9 ± 1.5	12.4 ± 0.7
	2	Radius	18.5	17	13.5	0	23.5	21	14.5	7
		Concentration	43.8 ± 1.8	38.8 ± 1.8	36.5 ± 2.6	≥48.5	14.2 ± 1.7	12.2 ± 2.3	13.8 ± 0.9	15.2 ± 1.0
*K. pneumoniae* ATCC 700603	0.5	Radius	14	11	8.5	2.5	15	12.5	7.5	2.5
		Concentration	53.1 ± 3.4	49.4 ± 1.9	45.3 ± 1.6	45.2 ± 1.7	19.9 ± 0.5	16.7 ± 0.6	16.3 ± 0.4	17.6 ± 0.6
	1	Radius	14	11.5	6	0	13.5	11	8	1.5
		Concentration	53.2 ± 4.0	48.8 ± 2.0	49.6 ± 2.5	≥48.5	20.9 ± 0.8	17.4 ± 0.7	16.1 ± 0.6	18.0 ± 0.7
	2	Radius	14	11	5.5	0.5	14	13	9	0.5
		Concentration	53.7 ± 3.7	49.4 ± 3.4	50.4 ± 2.9	≥48,5	20.7 ± 0.4	16.5 ± 0.6	15.8 ± 0.6	≥17.9
*E. faecalis* ATCC 29212	0.5	Radius	17.5	15	10.5	0.5	22	19	10	1.5
		Concentration	46.2 ± 4.9	42.2 ± 3.6	41.3 ± 4.6	≥48.5	15.2 ± 0.5	13.2 ± 1.7	15.5 ± 1.1	18.0 ± 1.3
	1	Radius	16.5	15	4	0	22	17	10.5	5.5
		Concentration	48.5 ± 1.8	42.8 ± 1.4	52.9 ± 7.7	≥48.5	15.4 ± 0.7	14.3 ± 2.0	15.3 ± 0.3	16.1 ± 3.5
	2	Radius	15.5	14	1.5	0	20	17.5	7	0
		Concentration	50.5 ± 2.0	44.3 ± 2.4	57.9 ± 4.7	≥48.5	16.7 ± 2.1	14.0 ± 1.6	16.5 ± 2.0	≥17.9
*S. aureus* ATCC 29213	0.5	Radius	22.5	19.5	15.5	14	28	26.5	25	15.5
		Concentration	36.1 ± 3.7	34.0 ± 2.9	32.8 ± 2.1	26.1 ± 2.0	11.3 ± 0.7	9.3 ± 0.5	10.4 ± 0.4	10.9 ± 2.6
	1	Radius	21	19	14.5	5.5	27.5	26	23.5	10.5
		Concentration	39.4 ± 1.9	35.4 ± 2.2	34.2 ± 1.8	40.6 ± 8.8	11.7 ± 1.0	9.5 ± 0.7	10.9 ± 0.5	13.5 ± 1.8
	2	Radio	19.5	17	12.5	6	25	26	14.5	9.5
		Concentration	42.3 ± 2.2	38.6 ± 1.9	38.0 ± 2.5	39.1 ± 6.1	13.3 ± 1.7	11.1 ± 0.7	13.8 ± 2.3	14.0 ± 1.1
*C. albicans* ATCC 200955	0.5	Radius	41	38.5	37	34.5	42	41	38.5	35
		Concentration	≤9.4	≤5.8	≤ 4.2	≤8.5	≤4.2	≤2.6	≤7.5	≤2.8
	1	Radius	39.5	38.5	34.5	32	42	40.5	39	36
		Concentration	≤9.4	≤5.8	≤4.2	≤8.5	≤4.2	≤2.6	≤7.5	≤2.8
	2	Radius	38	37.5	35.5	31	40.5	38	35.5	33.5
		Concentration	≤9.4	≤5.8	≤4.2	≤8.5	≤4.2	≤2.6	≤7.5	≤2.8
*C. krusei* ATCC 6258	0.5	Radius	43	41.5	40.5	36.5	43.5	43.5	41	38.5
		Concentration	≤9.4	≤5.8	≤4.2	≤8.5	≤4.2	≤2.6	≤7.5	≤2.8
	1	Radius	42.5	42	37.5	35.5	41.5	42	38	36.5
		Concentration	≤9.4	≤5.8	≤4.2	≤8.5	≤4.2	≤2.6	≤7.5	≤2.8
	2	Radius	42	36	37	34	41	39.5	37.5	36
		Concentration	≤9.4	≤5.8	≤4.2	≤8.5	≤4.2	≤2.6	≤7.5	≤2.8

Radius: average value (in mm) of the radius of the growth inhibition halos for the 10 assays carried out with each microorganism, inoculum, concentration and organosulfur compound (see Table 2).

## Data Availability

The data presented in this study are available in the main text.
